# A systematic review of hybrid brain-computer interfaces: Taxonomy and usability perspectives

**DOI:** 10.1371/journal.pone.0176674

**Published:** 2017-04-28

**Authors:** Inchul Choi, Ilsun Rhiu, Yushin Lee, Myung Hwan Yun, Chang S. Nam

**Affiliations:** 1 Edward P. Fitts Department of Industrial and Systems Engineering, North Carolina State University, Raleigh, North Carolina, United States of America; 2 Division of Global Management Engineering, Hoseo University, Asan, Korea; 3 Department of Industrial Engineering, Seoul National University, Seoul, Korea; National University of Defense Technology College of Mechatronic Engineering and Automation, CHINA

## Abstract

A new Brain-Computer Interface (BCI) technique, which is called a hybrid BCI, has recently been proposed to address the limitations of conventional single BCI system. Although some hybrid BCI studies have shown promising results, the field of hybrid BCI is still in its infancy and there is much to be done. Especially, since the hybrid BCI systems are so complicated and complex, it is difficult to understand the constituent and role of a hybrid BCI system at a glance. Also, the complicated and complex systems make it difficult to evaluate the usability of the systems. We systematically reviewed and analyzed the current state-of-the-art hybrid BCI studies, and proposed a systematic taxonomy for classifying the types of hybrid BCIs with multiple taxonomic criteria. After reviewing 74 journal articles, hybrid BCIs could be categorized with respect to 1) the source of brain signals, 2) the characteristics of the brain signal, and 3) the characteristics of operation in each system. In addition, we exhaustively reviewed recent literature on usability of BCIs. To identify the key evaluation dimensions of usability, we focused on task and measurement characteristics of BCI usability. We classified and summarized 31 BCI usability journal articles according to task characteristics (type and description of task) and measurement characteristics (subjective and objective measures). Afterwards, we proposed usability dimensions for BCI and hybrid BCI systems according to three core-constructs: Satisfaction, effectiveness, and efficiency with recommendations for further research. This paper can help BCI researchers, even those who are new to the field, can easily understand the complex structure of the hybrid systems at a glance. Recommendations for future research can also be helpful in establishing research directions and gaining insight in how to solve ergonomics and HCI design issues surrounding BCI and hybrid BCI systems by usability evaluation.

## Introduction

### Background and motivation

During the last decades, a new technology, called a brain-computer interface (BCI), has emerged by allowing the human brain to directly communicate with the environment. The BCI, also known as a brain-machine interface or a direct neural interface, is a non-muscular communication system that does not depend on the brain’s normal output pathways of peripheral nerves and muscles, so it can provide a direct connection from the brain to communicate and control devices [1]. Many BCI research projects have been produced to provide alternate methods to interact with the outside world not only for healthy people [2–4], but also for patients who cannot use their muscles due to an injury or a disease but are cognitively intact [5–7]. These BCI studies found many useful applications for the technology, and have been validated by target users with valuable and promising results [8–14].

Despite these advances and a considerable amount of ongoing research, current efforts in the area of BCI research and development have uncovered significant gaps, ‘universality’ [15,16] and ‘non-stationarity’ [17–19]. First, in BCIs based on imagined movements approximately 20% of users do not exhibit BCI performance adequate enough for effective control, a phenomenon called ‘BCI illiteracy’ [2,15,20–23]. This problem has been reported with other major BCI approaches as well. For instance, some studies which explored universality with P300 and steady-state visual evoked potential (SSVEP) BCIs found that these approaches may work for a larger percentage of users, but not necessarily all of them [16,20,24,25]. Second, brain signal patterns vary within a subject over time as well as between individuals, and this non-stationarity makes it difficult to decode the brain signal properly and results in extensive training being needed to effectively use the devices [18,19]. Various methods have been proposed and applied to make BCIs more universal and to address non-stationarity, such as improved training [26,27], distributing proper instructions to the end users [28], and improved signal processing [29]. However, even with these new techniques, some of users still cannot use a resulting BCI [15,21,30].

Recently, novel approaches have been proposed to address these issues in current BCI studies by combining a BCI system with other system(s) that utilize neurological signals, physiological signals, and/or external signals. This new BCI technique is called a hybrid BCI or hBCI [4,31–34]. Since each system in the hybrid BCI capable of applying different signal acquisition methods with various signal features, hybrid BCIs consist of diverse input signals. For example, input signals used in hybrid BCIs can be (1) two of the same types of brain signal (e.g., two electroencephalographies, EEGs), (2) two different types of brain imaging methods (e.g., EEG and functional Near-Infrared Spectroscopy, fNIRS), (3) one brain signal (e.g., EEG) and another physiological signal (e.g., heart rate or HR variability), or (4) one brain signal (e.g., EEG) and another conventional input (e.g., eye tracker). Furthermore, various hybrid BCI systems have been constructed sequentially or simultaneously by combining one BCI system with another system that is or is not BCI-based. In this case, each system in the hybrid paradigm can have different roles such that one system can be used as a switch to initiate or stop another system [31,35,36], both system can perform together for a common goal by supporting each other [20,37,38], or each system has different goals such as two-dimensional control [39,40]. Some of these hybrid BCIs have been shown to reduce disadvantages of each conventional BCI system so that the first BCI might be feasible for users who cannot use the second BCI, and vice versa. Furthermore, hybrid BCIs can increase accuracy and Information Transfer Rate (ITR). For example, Allison et al. [20] combined Event-Related Desynchronization (ERD) and SSVEP features into a hybrid BCI, in which subjects imagined the left (or right) hand movement while attending to the left (or right) flickering LED at different frequencies. The authors found that compared to the ERD and the SSVEP BCI condition the hybrid BCI (1) improved classification accuracy, (2) reduced BCI illiteracy, and (3) had a level of workload comparable to the ERD and the SSVEP BCI. Similarly, Combaz and Van Hulle [38] combined SSVEP and P300 brain signals to improve ITR by increasing the speed of BCI performance and the number of targets. Scherer and colleagues [31] used heart rate responses to initiate a BCI-controlled prosthetic under an asynchronous paradigm. Furthermore, Yin et al. [41] combined two brain signals from EEG and fNIRS systems to improve the performance of decoding motor imagination by utilizing advantages of each brain imaging method. Finally, Lim et al. [35] utilized SSVEP with eye tracking data to prevent errors from SSVEP-based BCI system.

Although some research studies have shown promising results using hybrid BCIs, the field of hybrid BCIs is still so new that no topic area within this domain can be considered mature. Although some areas in BCIs have been explored more extensively, the BCI community is only recently beginning to understand how to develop and control certain aspects of hybrid BCIs. Since its early research in hybrid BCIs, the field has made much progress and in a short time, with a much wider variety of hybrid BCIs being developed. However, there are two issues that should be addressed in order to achieve similar or better results in the forthcoming years.

The first issue of the current research studies is that the complicated and complex system structure of the hybrid BCIs. As mentioned previously, the hybrid BCI system consists of at least one system utilizing a neurological signal combined with other interface(s) using neurological, physiological, and/or an external signal. Furthermore, each system could have a different role of operation such as complementary cooperation for a common goal, independent duties for separate goals, an on-off switch, and a selector. Due to intricate combinations, it becomes hard to understand the constituent and role of a hybrid BCI system at a glance. Some researchers have tried to address this issue by applying taxonomic criterion to classify different hybrid BCI types. Pfurtscheller et al. [34] classified hybrid BCIs with respect to different processing types such as sequential and simultaneous BCIs. Severens et al. [42] categorized hybrid BCIs with different brain signal measurement types, sensory types (modality), and neurophysiological response types (signature). Allison et al. [4] named a hybrid system that included two BCI systems as ‘a pure hybrid‘; one BCI system with other physiological systems as ‘a physiological hybrid‘; and one BCI system with other external systems as ‘a mixed hybrid‘. However, these definitions and terminologies are not consistent among hybrid BCI studies, and some studies were misclassified as hybrid BCI systems which were actually not hybrid BCIs. Furthermore, some researchers have tried to propose taxonomical approaches for some features, such as a role of operation and signal types to classify hybrid BCIs, but there are no comprehensive review studies that can provide a clear and systematical taxonomy of hybrid BCIs with multiple taxonomic criteria as of the publication of this paper. These issues make it difficult to understand the structure of hybrid BCI studies, as well as select effective hybrid BCI systems according to target users and goals of application [43–46].

The other issue is that complicated and complex hybrid BCIs make it difficult to evaluate the system in terms of usability. Furthermore, additional communication channels and extra features can increase interactions between users and systems, but it could also cause negative effects on the user performance and satisfaction due to high mental workloads [47–50]. Recently a tremendous amount of studies related to usability in the field of Human-Computer Interaction (HCI) have been published and as such researchers in BCI agree with these findings that usability is an indispensable quality of BCI systems [51–56]. From this point of view, several researchers are trying to conduct usability evaluations of BCI systems. For example, BCI systems are mainly evaluated in the perspective of efficiency, such as classification accuracy and communication speed [57]. Usability dimensions, including efficiency measures, workload and satisfaction, have been assessed by Riccio et al. [58] and Zander & Gaertner [59]. Pasqualotto [60,61] investigated error rate and learnability of keyboard-controlled BCI prototypes. However, there were some notable limitations in these previous studies. First, many studies have only been focused on the performance measurement, such as accuracy and ITR [62–66]. Since very few studies have been made on heuristic evaluation for usability of BCI systems, it is difficult to determine if the proposed BCI systems are easy to use for everyone. Second, there is no well-structured usability framework which has a wide application for BCI studies. As Charlton and O’Brien [67] maintained, evaluations are not always systematically planned, but are often conducted based on the preference of the evaluator without careful considerations of various issues of usability evaluation. This is likely to result in irrelevant or useless results, and the evaluation efforts may turn out to be inefficient and unstructured. Essentially, a practical support is required regarding the measurement of usability. A widely accepted definition of usability implies that it should be measured in terms of effectiveness, efficiency, and satisfaction [68]. It often gets fuzzy when the practitioners need to figure out exactly what measures are representative for these three aspects. In the case of hybrid BCI systems, which utilize multiple biological signal or some traditional input devices, it is difficult to use the results of previous studies related to usability of other devices including BCIs due to varying and complex user experiences. Thus, a new framework is required for the hybrid BCI usability evaluation methodology, which should be simple and useful to practitioners as well as experts who conduct usability evaluations.

### Review objectives

This review study was designed to present and analyze the current state-of-the-art hybrid BCIs backed up with an elaborated taxonomy of hybrid BCIs that can provide additional insight into the design space, as well as analytical and experimental comparisons. This study also aimed to provide an in-depth discussion of usability in hybrid BCIs, along with an outline of potentially useful approaches to tackle the challenges identified. To achieve these goals, we investigated following two research questions based on these dimensions of analysis:

#### RQ1. What are the key criteria to establish a taxonomy of hybrid BCIs?

- To provide a structural methodology for categorizing the current hybrid BCI studies
- To clarify the current research limitations for future research directions

To address RQ1, we summarized and clarified the criteria, such as brain signal measurement types, role and mode of operation, strategy, signature, and modality of BCI systems which were used in the previous studies to categorize the distinct characteristics of hybrid BCIs. Based on the literature review, we proposed a taxonomy of hybrid BCIs to provide a structural methodology to categorize the current hybrid BCI studies with respect to 1) diversity of input signal, 2) role of operation, 3) mode of operation, 4) mental strategy, 5) brain signal signature, and 6) stimulus modality. The proposed taxonomy, which includes the most important features in a BCI system can help BCI researchers 1) classify hybrid BCIs systematically, 2) understand hybrid BCI studies at a glance, and 3) choose effective hybrid BCI types according to application goals [43–46]. Afterwards, we presented the grounds for an argument which elaborates on which combinations would be appropriate for certain environments and conditions based on the advantages and disadvantages of each hybrid BCI system. The proposed taxonomy can also clarify the current research limitations for future research directions.

#### RQ2. What are the key evaluation dimensions of usability in BCI usability studies?

- To classify and summarize studies related to BCI usability according to task and measurement characteristics
- To drive suggestions to evaluate usability of hybrid BCIs

To address RQ2, we categorized BCI usability studies according to task and measurement characteristics. From the results of classification, we proposed usability dimensions for BCI systems. Then, we suggested further research related to usability evaluation of BCI and hybrid BCI systems considering ergonomic issues of BCIs and hybrid BCI systems. The proposed usability dimensions can help researchers and practitioners 1) understand BCI studies related to usability evaluation, 2) choose proper metrics for usability evaluation, and 3) evaluate usability of hybrid BCIs as well as general BCIs. Also, suggestions of future research directions in this study can be helpful in establishing research directions and gaining insights under the perspectives of ergonomics.

The remainder of this paper is organized as follows: Study 1 described an elaborated taxonomy based on the systematic literature review, followed by categorized current research studies according to the proposed taxonomy. Study 2 presented usability evaluation metrics for BCIs and hybrid BCI systems. Finally, the following sections explained opportunities for further research of hybrid BCIs, and presented the major conclusions.

## Study 1: Taxonomy of hybrid BCIs

### Search methodology

In this review, a systematical approach, called the Preferred Reporting Items for Systematic reviews and Meta-Analyses (PRISMA) [69], was utilized (see S1 Checklist). Articles were sought out from five major search engines including IEEE Xplore, PubMed, Engineering Village, Web of Science, and Scopus, since those engines cover engineering and medical topics, as well as a broad-spectrum perspective [70]. Eligibility and exclusion criteria follow.

#### Information sources

Various online databases were searched in this study: (1) IEEE Xplore to provide an electrical/electronic engineering perspective, (2) PubMed to provide a medical perspective, (3) Engineering Village to provide an engineering perspective, (4) Web of Science to provide a cross-disciplinary perspective, and (5) Scopus to provide a broad-spectrum perspective.

#### Inclusion and prescreening criteria

**Fig 1:**
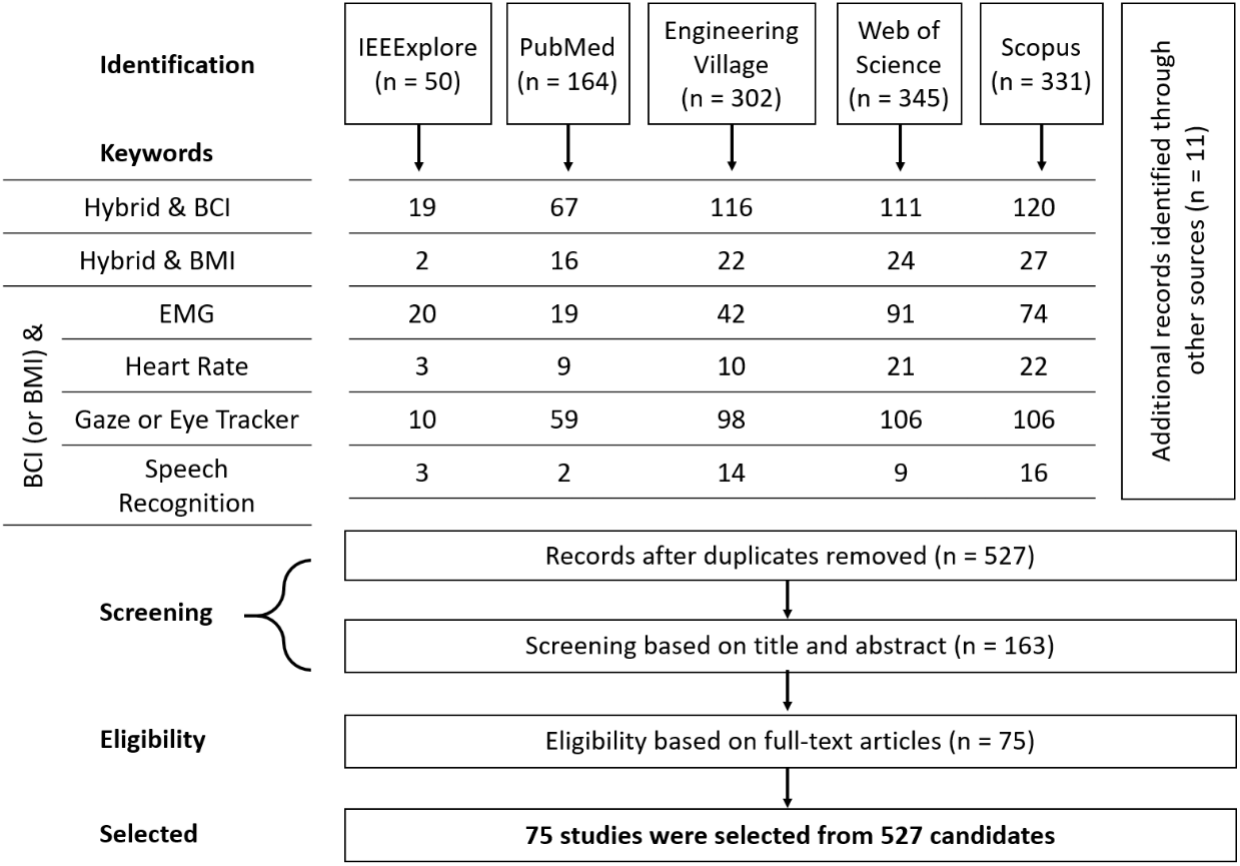
PRISMA flow diagram of hybrid BCIs.

Inclusion criteria were journal articles written in English from 2007 to 20 December 2016, since the first journal article related to the hybrid BCI system was published in 2007 [31,32]. Other publication forms (e.g., proceeding papers, unpublished working papers, master’s and doctoral dissertations, newspapers, and books, etc.) were not included. Since journal articles indicate a high level of research, journal articles can help both practitioners and academicians to obtain knowledge and spread their study findings. Keywords used in search engines were 1) “Hybrid” and “Brain computer interface”, 2) “Hybrid” and “Brain machine interface”, 3) combinations of either “Brain computer interface” or “Brain machine interface” with “electromyography,” “heart rate”, gaze, “eye tracker”, and “speech recognition”. After conducting the keyword search, 50, 164, 302, 345, and 331 articles were found from each search engine respectively, and then addition records were identified through other sources. Fig 1 shows the combinations of keywords and the number of studies for each keyword combination from the online databases. The total number of papers in each search engine was smaller than the summation of each keywords as shown in Fig 1, because some papers were found more than one time with different keywords. For example, the paper titled “Quantitative evaluation of a low-cost noninvasive hybrid interface based on EEG and eye movement” [71] was searched with keywords “hybrid & brain computer interface”, as well as with keywords “brain computer interface & eye tracker” in the IEEExplore. After the keyword search, duplicates were removed, and 527 articles remained. Those articles were screened based on titles and abstracts which were related to hybrid BCI topics, and 163 research studies were remained.

#### Eligibility criteria

Prescreened articles were checked for the eligibility via full-text screening by following analyses of populations, interventions, comparisons, outcomes, and study design (PICOS) [69]:

- **Populations**: Studies conducted with human subjects for any age, gender, or clinical conditions met the inclusion criteria, but any studies with non-human subjects, such as primates, were excluded.
- **Interventions:** All non-invasive hybrid BCI systems including at least one BCI system satisfied the eligibility.
- **Comparators**: Any study conditions such as multiple groups, single group, or case studies, were considered, because the main objective of Study 1 was proposing a hybrid BCI taxonomy method.
- **Outcomes**: All studies including classification procedures to achieve the main goal of BCIs met the inclusion criteria. However, some neuroscience studies that did not include classification results, but only examined the characteristics of the brain signal were excluded.
- **Study designs**: Any study designs had applied multiple systems including at least one BCI system were selected for further review.

### Search results and discussion

#### Taxonomic criteria for hybrid BCIs

After eligibility screening, 75 studies remained for the review from the initial 527 candidates. The selected journal articles were utilized to investigate important BCI features for taxonomic criteria of hybrid BCIs, and to propose a novel hybrid BCI taxonomy method (displayed in the following sections). Afterwards, the characteristics of each type, such as advantages and disadvantages, experimental environment, and applications, were discussed. Fig 1 shows the flow diagram of PRISMA with the results of keyword searching.

From the full-text review of articles, the following BCI features were found:

- Diversity of input signal: Single brain signal, multiple brain signals, brain signal(s) with physiological signals, and brain signal(s) with other signals from external devices
- Mental strategy: Selective attention and operant conditioning
- Stimulus modality: Visual, auditory, tactile, and operant conditioning
- Brain signal signature: Transient, steady-state, and different cognitive efforts
- Role of operation: Simultaneous and sequential
- Mode of operation: Synchronous and asynchronous

Based on the selected BCI features, hybrid BCI systems can be classified in terms of 1) the source of the signals, 2) the characteristics of the signal, and 3) the characteristics of operation in each system. Thus, each feature will be used as a taxonomic criterion in the following sections, and be utilized as a basis for the proposed taxonomy of hybrid BCIs. Besides the aforementioned features, brain signal recording methods, also known as brain imaging methods, are also an important BCI feature. Brain imaging methods can be categorized as non-invasive methods including EEG, fNIRS, and functional magnetic resonance imaging, and invasive methods including electrocorticography and intracortical neuron recording. In this review, only non-invasive methods were discussed due to limited applications of invasive technologies requiring surgical interventions [11].

#### Diversity of input signal

In the hybrid BCI paradigm, a brain signal can be combined with other brain signal(s), physiological signal(s), or external signal(s). For example, two or more brain imaging methods can be combined in a hybrid BCI such that brain signals from an EEG and fNIRS system in order to take advantage of each brain imaging technology [41,72–75]. Some research studies applied other physiological signals, such as electromyography (EMG) [32,76], electrooculogram (EOG) [77,78] and electrocardiography (ECG) [31,79] to brain signal(s) to address common limitations of brain signals, such as lower amplitude, non-stationarity, and vulnerability to muscle artifact. In addition, external signals can be added to support BCI systems including eye-tracking [71,80], a gyroscope [81], a position sensor [82], and a joystick [83,84]. Single BCI systems using one brain signal can also be classified into hybrid BCIs by combining two brain signal signatures such as Event-related Potential (ERP) and sensorimotor rhythm (SMR) induced by Motor Imagery (MI) [85,86], ERP and Steady State Evoked Potential (SSEP) [87,88], and SMR and SSEP [89,90]. The different brain signal signatures in the hybrid BCI will be discussed in the following section.

**Fig 2:**
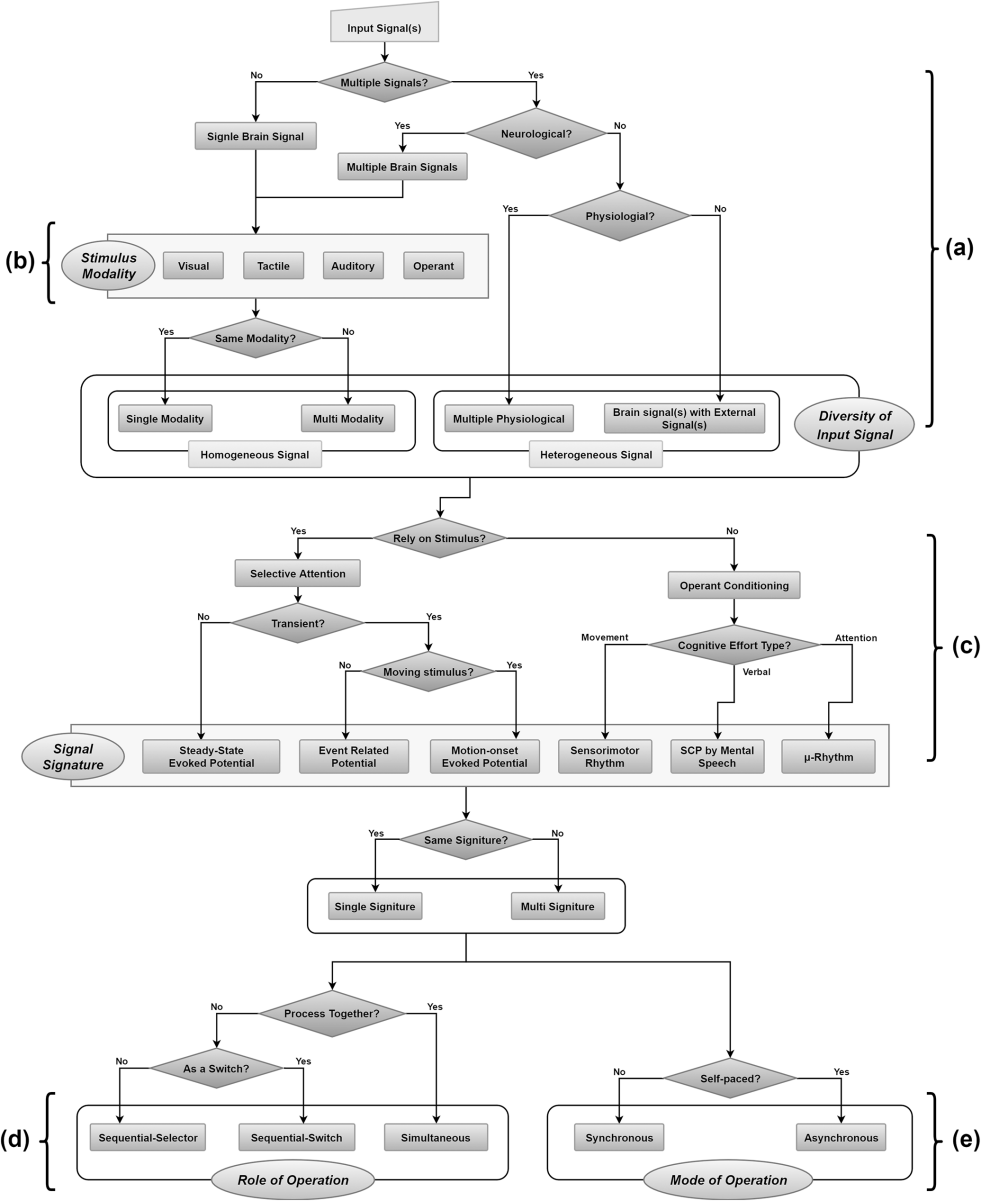
Flow diagram of taxonomy for hybrid BCIs.

In this review, a hybrid system that combined a brain signal with other brain signal(s) was defined as a homogeneous hybrid BCI system, while one combined with other physiological signals (non-neurological), or external signals was defined as a heterogeneous hybrid BCI system in terms of the signal diversity. Fig 2(A) illustrates a flow diagram to categorize hybrid BCIs with respect to different signal types.

Characteristics of Different Input Signal: As defined in the previous section, hybrid BCIs can consist of multiple brain signals, brain signal(s) with other non-neurological signal(s), or even a single brain signal. Firstly, the advantages of a single-brain signal approach are simple and easy to measure with a single brain imaging device [38,40]. Meanwhile, a multi-brain signal acquisition approach can resolve the inherent limitations of individual brain imaging methods [41]. For example, EEG and fNIRS could be used complementary to one another to measure brain signal features, because EEG has high temporal resolution and low spatial resolution while fNIRS has high spatial resolution and low temporal resolution [41,72–75]. A multi-physiological acquisition method has the advantage of higher classification accuracy due to not only the application of the classification result with additional physiological signals, but also the high signal-to-noise ratio of EMG and EOG signal [32,76–78]. Contrary to the combinations of physiological signals, external inputs including joysticks, eye trackers, and gyroscopes are directly utilized as a controller by modulating hand or body movements for directional applications such as a navigation [59], robot control [91] and game control [83]. However, multiple physiological signals and external inputs have limitations on the usage because of the need for physical movement, which can lead to electrode drift noise and muscle artifact on brain signals [32]. Table 1 shows the combinations of input signals and the number of studies for different diversities of input signals, and as shown in the table, most of the previous hybrid BCI research has been studied using a single-brain signal; and multiple physiological signals, combined with external inputs, and multiple brain signals follow in the order.

**Table 1:** The number of studies with each combination of biosignal.

Type	Input Signal		# of Studies
Single Brain Signal	EEG	EEG	44 (59%)
Multiple Physiological Signals	EEG	EOG	6 (8%)
	EEG	EMG	3 (4%)
	EEG	ECG	2 (3%)
Brain Signal with External Input	EEG	Eye Tracking	11 (15%)
	EEG	Joystick	2 (3%)
	EEG	Gyroscope	1 (1%)
Multiple Brain Signals	EEG	fNIRS	6 (8%)

Experiment Paradigm and Applications: The experimental environment varies with respect to a goal of research studies. The studies using the single brain signal and multiple brain signals cover general goals of BCI research. However, the research studies utilizing multiple physiological signal and external inputs usually involve physical movements including hand, head, and eyeball movements either to detect subject status or to improve the BCI performance. For example, Li and Chung [81] analyzed EEG signals for different attention levels and head-movement for yawning and rubbing face to detect a driver’s drowsiness in a driving simulation environment. Ma et al. [77] used eye-movements including blinking, frowning, winking, and gazing, to select a target action between different actions of robots by analyzing EOG signals, while EEG signals were utilized to control a robot according to the selected action. On the other hand, Park & colleagues [92] distinguished between navigational intentions (searching images to obtain the information) and informational intentions (finding a predefined target from images) by analyzing eye tracking data and EEG signal, and the authors reported that the classification accuracy of combining eye movement and EEG features showed higher accuracy than that of the eye movement feature and EEG features alone (90.9%, 85.8%, and 83.9%, respectively). From the literature review, we found that the role of each signal varies in experimental conditions, and the physiological signals were usually utilized as a selector or switch to support the neurological signal [76,93,94] while external inputs utilized direct control [83,84].

#### Mental strategy, signal signature, and stimulus modality

Brain signal can be either evoked by a stimulus or modulated by operant conditioning with respect to mental strategy [32,82,83]. Stimulus evoked brain signal requires selective attention on stimulus such as visual, tactile, and auditory modalities. On the contrary, operant conditioning does not depend on an external stimulus, but can be modulated by operant modalities, such as movement related efforts, attention, mental speech, and memory tasks.

In selective attention, stimuli can be categorized into either steady-state, or transient signature. The former can evoke SSEPs [89,90], and the latter elicits either ERPs or motion-onset (visual) evoked potentials (mVEPs) with different sensory modalities [39,80,95,96]. The mVEP signature was first employed by Guo et al. [97], and the motion-onset VEP-based BCI has the advantages of less visual fatigue and discomfort compared to other visual-based BCI systems [98]. For operant conditioning, Slow Cortical Potential (SCP) signatures can be modulated via different operant modalities. However, many researchers have differentiated SCP modulated by mental tasks from either movement related efforts or different attention levels elicited brain signal patterns [99–101]. The former is classified as an SMR signature evoked by movement related efforts, while the latter is known as a μ-rhythm signature. The movement related efforts include motor execution, movement attempt, and MI. Motor execution indicates actual a physical movement [81,102,103], while MI is mental movement imagination [40,89,90]. Movement attempt is a special case of motor execution only occurring during a motor attempt that involves paralyzed body parts [82,104]. Also speech and music imageries can be decoded to different brain signal signatures [79,105], and these brain signal signatures were categorized as SCP in this review. Fig 2(C) illustrates the flow diagram used to categorize hybrid BCIs with respect to different mental strategies and brain signal signatures.

In the hybrid BCI paradigm, either one stimulus or multiple stimuli with respect to sensory pathway such as visual (e.g., Steady-State Visual Evoked Potential or SSVEP), tactile (e.g., Steady-State Somatosensory Evoked Potential or SSSEP), and auditory (e.g., steady-state auditory evoked potential) modalities can be utilized. Similarly, ERP, also known as P300, can include visual, tactile, and auditory stimuli. If the hybrid BCI includes SSVEP and visual P300, then this hybrid BCI system is categorized as single modality [38,106]. On the contrary, if the hybrid BCI consists of SSVEP and SSSEP, then this system has multi-modality. In this review, operant conditionings including cognitive efforts, MI, and μ-rhythm, also belong to stimulus modality, because these conditionings can be deal with internal stimulus elicited by mental tasks. Fig 2(B) shows the flow diagram for categorizing hybrid BCIs with respect to stimulus modalities.

Characteristics of Different Strategies, Signatures, and Modalities: There are two main mental strategies including the selective attention such as SSEP and ERP, and operant conditioning such as SMR, SCP, and μ-rhythm [107]. Since selective attention only requires either focusing on continuous stimuli or counting transient events, the advantages of selective attention are 1) it is easier to perform BCI tasks, 2) a shorter training time is required, and 3) a higher classification accuracy especially with visual stimulus than operant conditioning exists [87,106]. However, this approach relies on external stimuli such as visual, tactile, and auditory. On the contrary, operant conditioning does not require any stimulus to evoke brain signal, but longer training periods are usually required to achieve reasonable classification accuracy [101,108]. The other possible advantage of operant conditioning with SMR-based BCI tasks is the neuroplasticity by facilitating motor-related brain area [109].

As each BCI modality has different characteristics, their advantages and disadvantages are also distinct [110]. Among different brain signal modalities, the advantages of visual modality are 1) higher classification accuracy and 2) easy to apply stimuli in the experimental environment by using either an LCD or LED [37,111,112]. However, since participants attend visual stimuli to evoke brain signals, they might feel annoyed, experience eye fatigue, and get even disturbance [77]. This issue could be addressed by combining mVEP with less visual fatigue and SMR without visual stimulation in multiple modalities and signatures [39]. The advantage of tactile and auditory modalities is the dependence of visual sensory, but there are some limitations of this application, such as lower classification accuracies and difficulties increasing the number of stimuli due to the nature of tactile and auditory sensory [75,89]. Operant modality such as MI is independent from visual stimulus but shows lower classification accuracy and needs longer training. For example, Allison et al. [111] utilized two different signatures and modalities in one EEG signal, SMR and SSVEP, for simultaneous two dimensional cursor control, and Combaz and Van Hulle [38] combined multiple signatures in a single modality, SSVEP and visual P300, to improve ITR. Yin et al. [41] measured both EEG and fNIRS brain signals to improve the performance of decoding SMRs evoked by MIs, while Lim et al. [35] combined SSVEP with eye tracking data to prevent errors in a SSVEP-based BCI system.

Experiment Paradigm and Applications: Visual-based BCIs are some of the more commonly studied BCI research studies because of the reliable results and short training time, and most of them are for a BCI speller [87,113,114]. Furthermore, Li et al. [115] proposed a multi-signature hybrid BCI system with visual P300 and SSVEP to control a wheelchair, while Pan et al. [88] to detect awareness of patients with disorders of consciousness in a similar BCI system. In addition, visual-based BCIs can be applied to games and virtual reality environments because visual stimuli can easily be embedded in these systems. There are also many SMR-based BCI studies that set out to control external devices such as a neuro-prosthesis, wheelchair, and exoskeleton [116,117], and the target users of these BCI systems are usually severely disabled patients. However, due to the limitations of SMR-based BCI systems discussed in the previous section, some SMR-based BCI research applied other modalities such as visual [34,86,118] and tactile [89,119] to increase the accuracy and robustness of the classification algorithms. Table 2 summarizes applications of each modalities with external signals braced with parentheses from the literature review.

**Table 2:** Applications of each modality in hybrid BCIs.

Applications	Modalities & External Signals		Studies
Mouse control	Visual	Operant: MI	[40] [86] [120]
Virtual environment	Visual	Operant: MI	[85] [121]
Wheelchair	Visual	Operant: MI	[117] [122]
	Visual	Visual	[115]
	(Eyeball)	Operant: MI	[78]
Email client	Visual	Operant: MI	[123]
Robot	Visual	Operant: MI	[118] [124]
	(Physical movement)	Operant: MI	[102]
	(Eyeball)	Operant: MI	[91]
	(Eyeball)	Visual	[77]
Neuroprosthetics	μ-rhythm	Operant: MI	[125]
	Visual	Operant: MI	[109] [126]
	(Physical movement)	Operant: MI	[82]
	(Eyeball)	Operant: MI	[116]
	(Heart rate)	Visual	[31]
GUI	Visual	Operant: MI	[94]
Driving simulation	(Physical movement)	Operant: Attention	[81]
Game	Visual	Operant: Attention	[101]
	(Physical movement)	Operant: MI	[83] [84]
Speller	Visual	Visual	[87] [106] [113] [114] [127] [128] [129]
	(Eyeball)	Visual	[35] [80] [95]
	(Physical movement)	Visual	[76]
	(Eyeball)	Operant: MI	[93]
Detecting awareness	Visual	Visual	[88]
Flight control	(Eyeball)	Operant: Attention	[100]
Navigation	Visual	Operant: MI	[59]
	(Eyeball)	Visual	[130]
	(Eyeball)	Operant: MI	[32]

#### Role and mode of operation

**Fig 3:**
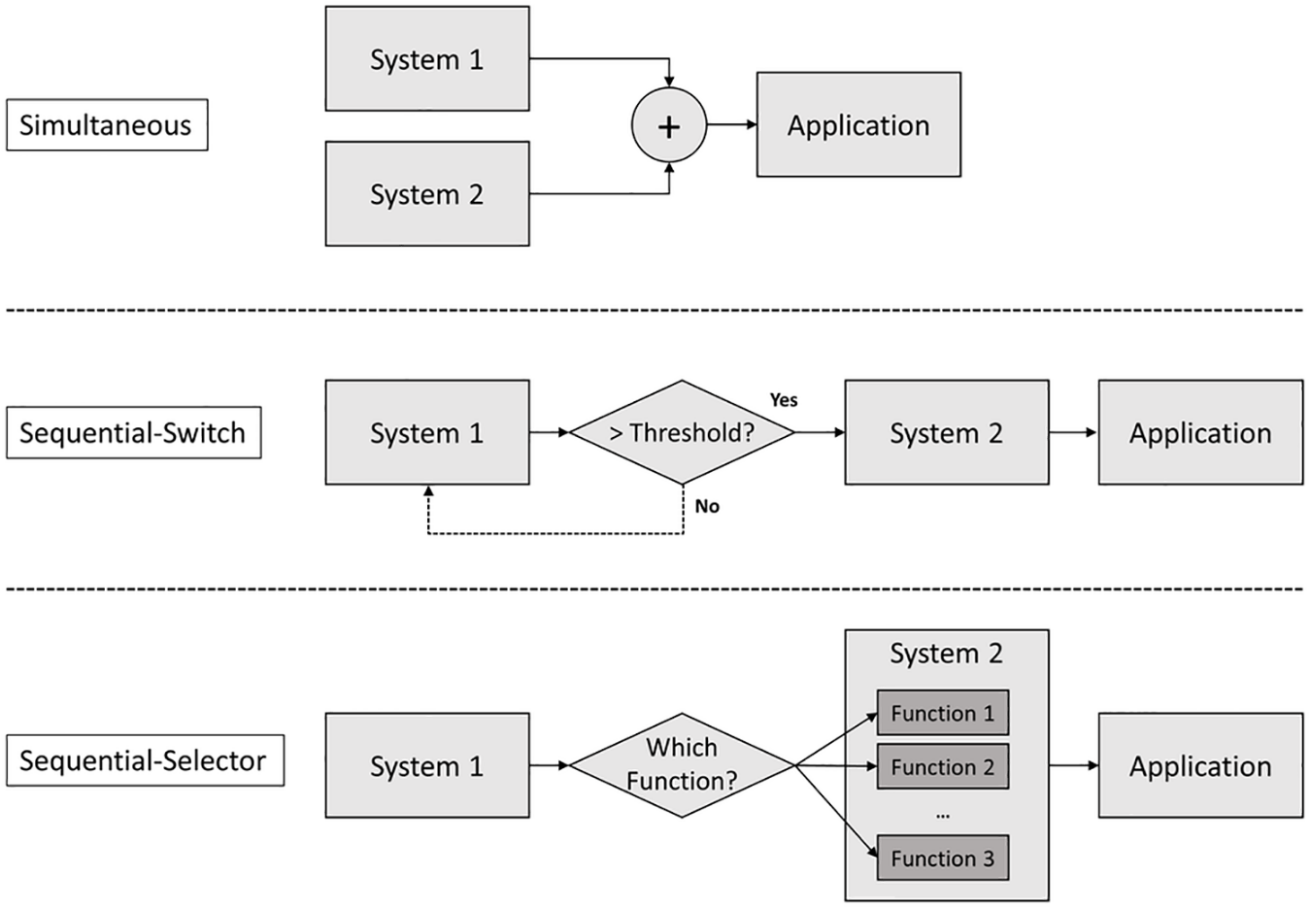
Schematic diagram for three different roles of operation.

A role of each system in the hybrid BCI can be different in terms of usages [34]. For example, both systems can play the same role simultaneously to achieve a certain goal. In this case, multiple input signals from different systems can be fed into one classification algorithm, or each decision can be fused to make one final decision. Yin et al. [113] utilized both SSVEP and visual P300 simultaneously to increase classification accuracy and ITR of a BCI speller, while Jiang et al. [131] fused MI features from EEG signals and gaze directions from EOG signals to improve the BCI performance for a multi-class target selection. It is also possible that one system can initiate the other system as a switch by detecting a distinct signal. For instance, SSVEP-based BCI can be used to turn an MI-based BCI on, then the MI-based BCI controls a hand orthosis to complete a hand grasping task [126]. Furthermore, both BCI systems can play different roles simultaneously to achieve different goals such as two-dimensional control [39,40,120]. For example, Ma et al. [39] combined SMR and mVEP signatures simultaneously to realize a 2-dimensionla cursor control, while Li et al. [120] utilized visual P300 and SMR signatures for the similar task. Finally, one system can decide to choose a certain function as a selector, then the other system can control levels in a specified sequence [124]. Fig 2(D) shows the flow diagram for categorizing hybrid BCIs with respect to the role of operation, and Fig 3 represents three different roles of operation.

Any BCI experiment falls into two different modes of operation. One mode of operation is defined as the BCI experiment being conducted under a synchronous, cue-paced scenario, and the other is under an asynchronous, self-paced manner. Synchronous experiments rely on a certain cue with a fixed time per trial, and the BCI systems control an application or provide feedback by analyzing brain signals of the fixed time [72,116]. On the contrary, experiments under asynchronous manner do not depend upon cues, but participants conduct BCI tasks towards a certain goal at their own pace [99,126].

Characteristics of Each Operation: The advantages of simultaneous processing are that 1) one-time signal processing is required, 2) multiple decisions can be made at one time, such as 2-demensional cursor control [120], and 3) the classification accuracy can be increased by facilitating two classification results complementarily. However, this approach also has limitations in applications of some BCI systems which involve multiple tasks occurring in a sequence such as sending an email [123] and controlling different levels between multiple functions [126]. However, hybrid BCI systems with a sequential processing mode can address these issues but this approach requires multiple BCI tasks to complete multiple steps.

Experiments under the synchronous mode are usually utilized to build parameters of classification algorithms [117,132]. Once the classifier is ready, participants can perform asynchronous experiments with goal-oriented tasks without cues. Therefore, the brain signal should be continuously monitored, and the BCI system will act only if the distinct signal is detected from the classifier during a series of tasks [102]. These two modes can be applied to any BCI system, and both modes have their own advantages and disadvantages discussed in the following section. Fig 2(E) illustrates the flow diagram for categorizing hybrid BCIs with respect to the mode of operation.

Experiment Paradigm and Applications: Both operation modes can be applied to many BCI applications. Xu et al. [87] applied P300 and SSVEP simultaneously for a BCI speller, and achieved higher accuracy and ITR than each BCI systems by combining two BCI features. Simultaneous BCI can be utilized for 2-demensional space control, and Allison et al. [111] showed promising results by apply MI for vertical movement and SSVEP for horizontal movement at the same time. Malechka et al. [94] proposed a BCI system with graphical User Interface (UI) to control activities of daily living applications by analyzing eye-tracker, SSVEP, and MI signals. In this system, participants select a device via eye tracker, select submenus such as volume and channel of radio by either SSVEP or MI. Kim et al. [100] built a BCI system to fly a drone in a real environment by using eye tracking and EEG data. EEG data was used to select two different modes, such as horizontal movements and vertical movements including turning. Afterwards, the results of the eye-tracker were used to control the drone. From the literature review, the applications of simultaneous mode were usually applied either to control two functions at one time or to increase classification accuracies and ITR. BCI systems using sequential processing were used either to control multiple functions with steps or to apply a switch function in asynchronous mode.

The experiments under the synchronous manner are useful to validate a proposed BCI system and to find user-specific classification parameters using offline analysis [117,132]. Since a synchronous BCI relies on external cues and a fixed BCI task time is required to perform signal processing, this approach has a practical limitation to apply in real-life tasks utilizing continuous controls [116]. This issue can be addressed by applying asynchronous techniques in which participants conduct BCI tasks towards a certain goal at their own pace without cues [99,126]. In this paradigm, the decisions are made in (near) real-time by classifying brain signals with classification parameters defined in the offline analysis. However, due to the non-stationarity of EEG signals [18], the classification accuracies under an asynchronous based system were usually lower than that of a synchronous approach.

Table 3 shows the classified hybrid BCI types from the selected 75 journal articles according to the proposed taxonomy, and non-neurological signals used in each study are braced with parentheses similar to Table 2.

**Table 3:** Classified hybrid BCI types according to the proposed taxonomy.

Article	Diversity of Input Signal			Role of Operation	Mode of Operation	Stimulus Modality		Signal Signature		# of Subject (# of Patient)
	Neurological		Others							
[20]	EEG			Simultaneous	Synchronous	Visual	Operant	SSVEP	SMR	14
[31]	EEG		ECG	Switch	Asynchronous	Visual		SSVEP		10
[32]	EEG		EMG, EOG	Simultaneous	Asynchronous	Operant		SMR		3
[37]	EEG			Simultaneous	Synchronous	Visual	Operant	SSVEP	SMR	14
[38]	EEG			Simultaneous	Synchronous	Visual	Visual	P300	SSVEP	9
[39]	EEG			Simultaneous	Asynchronous	Visual	Operant	mVEP	SMR	6
[40]	EEG			Simultaneous	Asynchronous	Visual	Operant	P300	SMR	11
[41]	EEG	fNIRS		Simultaneous	Synchronous	Operant		SMR		6
[42]	EEG			Simultaneous	Synchronous	Tactile	Tactile	SSSEP	P300	13
[59]	EEG		Eye tracking (Eyeball)	Selector	Asynchronous	Operant		SMR		10
[71]	EEG		Eye tracking (Eyeball)	Selector	Asynchronous	Operant		μ-rhythm		10
[72]	EEG	NIRS		Simultaneous	Synchronous	Operant		SMR		14
[73]	EEG	fNIRS		Switch	Asynchronous	Operant		SMR		6
[74]	EEG	NIRS		Simultaneous	Synchronous	Operant		SMR		14
[75]	EEG	fNIRS		Simultaneous	Synchronous	Visual	Auditory	ERP		12
[76]	EEG		EMG (Wrist movement)	Selector	Synchronous	Visual		P300		11 (3)
[77]	EEG		EOG (Eyeball)	Switch	Asynchronous	Visual		P300		13
[78]	EEG		EOG (Eyeball)	Switch	Asynchronous	Visual	Operant	P300	SMR	9
[79]	NIRS		ANS (EDA, ST, HR, and RE)	Simultaneous	Synchronous	Operant		SCP(Music Imagery)		8
[80]	EEG		Eye tracking (Eyeball)	Selector	Synchronous	Visual		P300		10
[81]	EEG		Gyroscope (Head movement)	Simultaneous	Asynchronous	Operant		θ, α, β		6
[82]	EEG		Position Sensor (Shoulder movement)	Switch	Asynchronous	Operant		SMR		1 (1)
[83]	EEG		Joystick	Simultaneous	Asynchronous	Operant	Operant	SMR	SMR	14
[84]	EEG		Joystick	Simultaneous	Asynchronous	Operant		SMR		10
[85]	EEG			Selector	Asynchronous	Visual	Operant	P300	SMR	4
[86]	EEG			Simultaneous	Asynchronous	Visual	Operant	P300	SMR	5
[87]	EEG			Simultaneous	Synchronous	Visual	Visual	P300	SSVEP	12
[88]	EEG			Simultaneous	Synchronous	Visual	Visual	P300	SSVEP	8 (8)
[89]	EEG			Simultaneous	Synchronous	Tactile	Operant	SSSEP	SMR	16
[90]	EEG			Simultaneous	Synchronous	Visual	Operant	SSVEP	SMR	24
[91]	EEG		Eye tracking (Eyeball)	Switch	Asynchronous	Operant		SMR		7 (4)
[92]	EEG		Eye tracking (Eyeball)	Simultaneous	Synchronous	Visual		SCP		8
[93]	EEG		Eye tracking (Eyeball)	Selector	Asynchronous	Operant		SMR		7
[94]	EEG		Eye tracking (Eyeball)	Selector	Asynchronous	Visual	Operant	SSVEP	SMR	6
[95]	EEG		EOG (Eyeball)	Simultaneous	Synchronous	Visual		P300		10
[96]	EEG			Simultaneous	Synchronous	Visual	Visual	P300	mVEP	10
[99]	EEG			Simultaneous	Asynchronous	Visual	Operant	SSVEP	μ-rhythm	6
[100]	EEG		Eye tracking (Eyeball)	Selector	Asynchronous	Operant		μ-rhythm		5
[101]	EEG			Selector	Synchronous	Visual	Operant	SSVEP	μ-rhythm	19
[105]	EEG			Simultaneous	Synchronous	Operant		SMR	Speech Imagery	7
[106]	EEG			Simultaneous	Synchronous	Visual	Visual	SSVEP	P300	10
[108]	EEG		Eye tracking (Eyeball)	Simultaneous	Synchronous	Operant		SMR		30
[109]	EEG			Selector	Asynchronous	Visual	Operant	SSVEP	SMR	6
[111]	EEG			Simultaneous	Synchronous	Visual	Operant	SSVEP	SMR	10
[112]	EEG			Simultaneous	Synchronous	Visual	Visual	P300	SSVEP	10
[113]	EEG			Simultaneous	Synchronous	Visual	Visual	P300	SSVEP	13
[114]	EEG			Simultaneous	Synchronous	Visual	Visual	P300	SSVEP	14
[115]	EEG			Simultaneous	Asynchronous	Visual	Visual	P300	SSVEP	8
[116]	EEG		EOG (Eyeball)	Simultaneous	Asynchronous	Operant		SMR		6 (1)
[117]	EEG			Simultaneous	Asynchronous	Visual	Operant	SSVEP	SMR	7
[118]	EEG			Selector	Asynchronous	Operant	Visual, ERN	SMR	P300, ErRP	5
[119]	EEG			Simultaneous	Synchronous	Tactile	Operant	SSSEP	SMR	11
[120]	EEG			Simultaneous	Synchronous	Visual	Operant	P300	SMR	10
[121]	EEG		Eye tracking (Eyeball)	Selector	Asynchronous	Operant		SMR		20
[122]	EEG			Switch	Asynchronous	Visual	Operant	SSVEP	SMR	3
[123]	EEG			Selector	Asynchronous	Visual	Operant	P300	SMR	6
[124]	EEG			Selector	Asynchronous	Visual	Operant	P300, SSVEP	SMR	5
[125]	EEG			Switch	Asynchronous	Operant	Operant	SMR	μ-rhythm	2 (2)
[126]	EEG			Switch	Asynchronous	Visual	Operant	SSVEP	SMR	6
[127]	EEG			Selector	Synchronous	Visual	Visual	P300	ErRP	12
[128]	EEG			Selector	Synchronous	Visual	Visual	P300	ErRP	12
[129]	EEG			Simultaneous	Synchronous	Visual	Visual	P300	SSVEP	12
[130]	EEG		Eye tracking (Eyeball)	Simultaneous	Asynchronous	Visual		ERP		10
[131]	EEG		EOG (Eyeball)	Simultaneous	Synchronous	Operant		SMR		4
[132]	EEG			Simultaneous	Synchronous	Visual	Operant	SSVEP	SMR	12
[133]	EEG			Selector	Asynchronous	Visual	Operant	P300	SMR	5
[134]	EEG		Position sensor (Shoulder movement)	Switch	Asynchronous	Operant		SMR		1
[135]	EEG			Simultaneous	Asynchronous	Visual		SSVEP		9
[136]	EEG			Selector	Synchronous	Visual	Operant	P300	SMR	12
[137]	EEG			Simultaneous	Synchronous	Auditory	Tactile	P300	P300	12
[138]	EEG			Selector	Synchronous	Auditory	Auditory	P300	ErRP	9
[139]	EEG			Simultaneous	Synchronous	Tactile	Tactile	P300	SSSEP	14
[140]	EEG		EMG (Hand movement)	Selector	Synchronous	Visual		SSVEP		10
[141]	EEG	fNIRS		Simultaneous	Synchronous	Operant		SMR		15
[142]	EEG			Simultaneous	Synchronous	Tactile	Operant	SSSEP	SMR	14

## Study 2: Usability evaluation metrics for hybrid BCIs

### Search methodology

For the systematic literature review, the PRISMA method was utilized similar to Study 1 [69]. A total of 279 articles dating from 2000 to January 2016 were obtained and reviewed (the first journal article related to BCI was published in 2000). Articles were found via computerized search. A detailed explanation of the methodology used for extracting articles follows.

#### Information sources and inclusion criteria

The online databases searched in Study 2 were identical to Study 1. Original studies that conducted usability evaluation on BCI with subjective measures or performance measures were included. This study covers only journal articles published in English. Other publication forms (e.g., proceeding papers, unpublished working papers, master’s and doctoral dissertations, newspapers, and books, etc.) were excluded.

Full-text review of articles includes the following analyses of populations, interventions, comparisons, outcomes, and study design:

- **Populations**: All populations were considered, but non-human subjects were excluded.
- Interventions: We screened for studies that measured the usability of BCI using subjective measures or performance measures.
- **Comparators**: We did not screen for studies that included the results of some kind of functional control comparison. Because our research goal focused on the investigation of usability measure we did not consider studies that included experiment designs with a control group or treatments.
- **Outcomes**: Studies were required to include objective/subjective measures of efficiency, effectiveness, satisfaction. For studies that did not explicitly include such a component, we screened for those studies the outcomes of which could be logically linked with usability.
- **Study designs:** Given the nature of BCI research, there is a narrow range of study designs employed. Most frequently we encountered small-n, within-subject designs. Thus, we did not screen for design type, beyond the requirement already stated that the design involve human subjects.

#### Search strategy and limits

As few studies related to usability evaluation of hybrid BCIs have been conducted, we focused on reviewing previous studies related to usability evaluation of BCI. Afterwards, the results of the review were utilized to suggest usability evaluation metrics for hybrid BCIs. Thus, the general search strategy included key terms such as “Brain computer interface”, and “Usability”.

### Search results

First, the five online databases were searched for articles in the same way as Study 1, then addition records were identified through reference lists of included articles. The total number of articles found was 317. The number of articles by each online database is as follows: Engineering Village (51), IEEE Xplore (8), PubMed (59), Scopus (84), and Web of Science (77). Next, manual removal of duplicate records excluded 165 records, yielding 152 unique articles for consideration. Examination of abstracts and titles excluded a further 98 articles, all due to not adequately conforming to any of our research questions, leaving 54 articles for full-text analysis. Full-text analysis of these articles excluded a total of 23 records, for the following reasons: three articles were eliminated for being an inadequate type of publication (e.g., review paper, conference paper); five articles were eliminated for not using any metrics to evaluate usability of BCI; and 15 articles were eliminated for not addressing our research question, or otherwise not meeting inclusion criteria.

**Fig 4:**
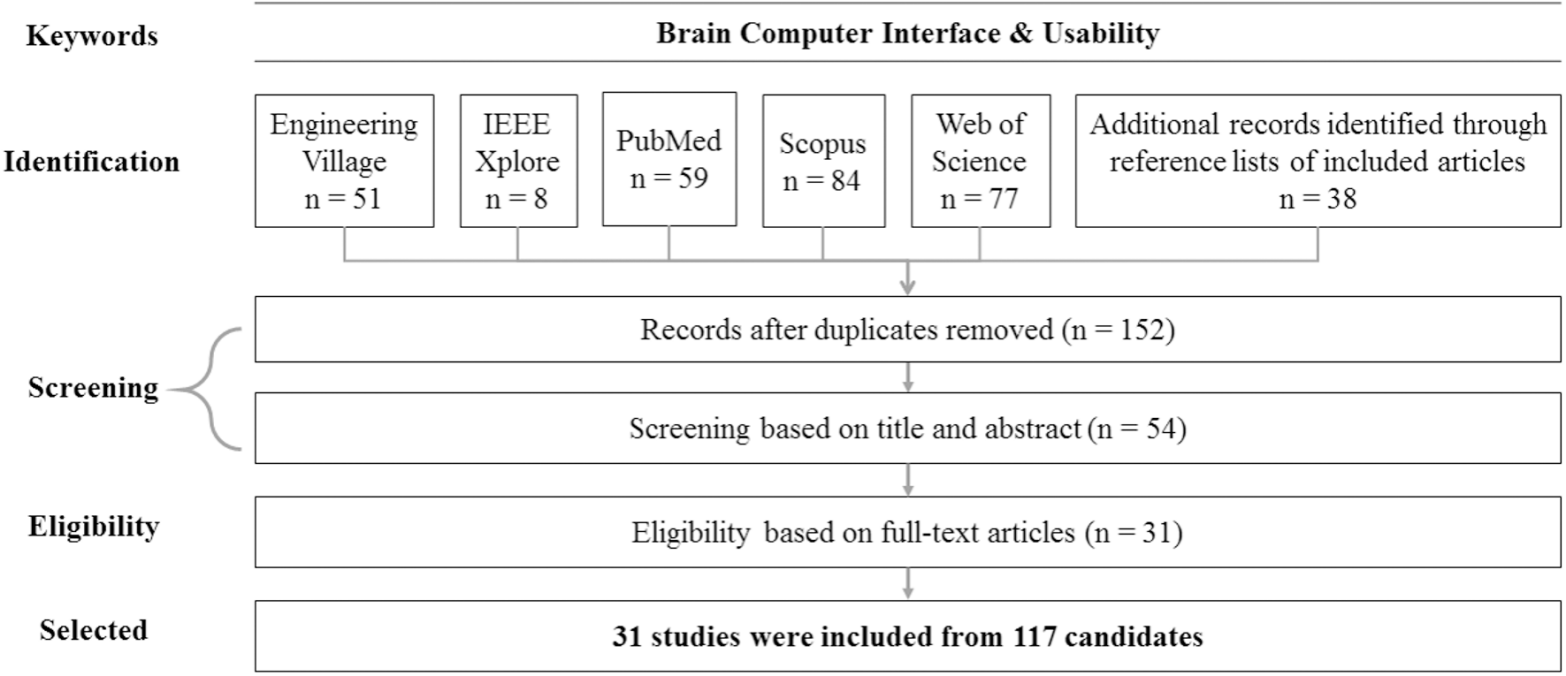
PRISMA flow diagram of usability of BCI.

Thus, a total of 31 articles for usability of BCI research met all the selection criteria. See Fig 4 for a PRISMA flow diagram summarizing the article review process.

### Study characteristics

#### Participants

Among the 31 studies that we reviewed, the average number of participants was 12.71, with a maximum of 39 and a minimum of 1. Some studies engaged a very small group of subjects because they targeted a population of disabled persons [52,143–145]. The age of participants varied depending on the study. Twenty out of 31 articles explicitly disclosed the age information of their subjects; one article did not mention participants’ age [62,146]. Based on the known information, the oldest participant was 73 years old [143], while the youngest one was 16 [147]. Subjects with disabilities tend to be older and subjects in the healthy group tend to be younger. Six articles reported the gender distribution of their participants [65,66,76,148–150]. Among most of them, the percentage of male subjects was more than 50%. Sixteen studies recruited participants with disabilities [55,65,66,76,143–145,148–156]. The most common type of disability was sclerosis [52,55,65,66,76,143–145,149–151,153–155]. Among them, two articles recruited multiple sclerosis patients, and nine articles recruited amyotrophic lateral sclerosis patients. Seven articles included disabled participants that had suffered from strokes [66,76,144,149,152,153]. Thirteen studies reported all healthy subjects [58,63,64,146,147,157–164], and one study did not disclose this information [62].

#### Study design

Most of the studies conducted were proof of concept design or within-subject design because of the limited number of patient-subjects. In the studies, researchers introduced their newly developed BCI system and evaluated its usability with or without existing systems. Only four studies used a between-subject design and they conducted experiments with only healthy subjects [147,157–159].

#### Study environment

Most of the experiments took place in a laboratory environment. Only three studies utilized a “daily life” environment (e.g, in the subject’s home) [143,152,155].

### Review results and discussion

Thirty-one articles were finally selected to address RQ2. These articles were categorized according to task characteristics and measurement characteristics respectively.

#### Task characteristics

Collected articles were categorized according to the type of task which is used for usability evaluation of BCI (See Table 4). The collected articles were categorized into seven tasks:

- Spelling: the task to type given or free words via BCI systems.
- Movement control: the task to control the movement of system.
- Selection control: the task to target and choose the icons or buttons.
- Brain painting: the task to utilize the painting program.
- Mental task: kind of thinking activity such as imagination, mental calculation and etc.
- Cognitive rehabilitation task: is kind of strengthening activity of intellectual capacity.

**Table 4:** Classification of articles by task characteristics.

Main categories	Sub categories	No. of articles	References
Type of task	Open task	3	[64,143,153]
	Closed task (self-managed)	9	[52,58,145,147,152,154,158,159,163]
	Closed task (copy)	21	[52,55,62,63,65,66,76,144–151,154,157,160–163]
Description of task	Spelling	19	[55,58,63,65,76,144–146,148–152,154,157,161–164]
	Control (moving)	9	[52,58,62,64,66,148,153,154,160]
	Control (selecting)	9	[52,58,64,149,154–156,158,159]
	Brain painting	2	[143,144]
	Mental task	2	[63,147]
	Cognitive rehabilitation task	1	[148]

According to the characteristics of the task goal, we divided tasks into two types: open task and closed task. If the user defines the outcome of the task, it is considered an open task. In the case of a closed task, the experimenter gives pre-defined goals to users [165]. For example, freely using the program is an open task experiment but using the program according to pre-defined instruction is considered a closed task design. Again, we categorized closed task experiments by the characteristics of strategy. If the user can freely choose the strategy to achieve the pre-defined goal, it is a closed self-managing task. If users have no choice and just follow the pre-defined strategy, it is a closed copying task. For example, typing words from the users’ own thought is considered a closed self-managing task but typing given words is a closed copying task.

In the BCI usability studies, spelling tasks were the most frequently used tasks. Control tasks (e.g., movement and control) were often used too. In order of frequency of use, brain painting task, mental task, and cognitive rehabilitation task were used. With consideration of type of task, closed tasks were dominantly used and closed copying task was more used than self-managing task. Type of open task is given only in the few studies which used movement control, selection control, and brain painting as a task. In studies involving the spelling and movement control task, closed copying task was used most frequently.

#### Measurement characteristics

Overall, 10 evaluation tools of subjective measures were used in 31 BCI usability studies (see Table 5). NASA Task Load Index (NASA-TLX), Visual Analogue Scale (VAS), Assistive Technology Device Predisposition Assessment (ATD-PA) device form, System Usability Scale (SUS) survey, Quebec User Evaluation of Satisfaction with assistive Technology 2.0 (QUEST 2.0), IBM’s computer usability satisfaction questionnaire and Usefulness, Satisfaction, and Ease of use (USE) questionnaire were utilized. Some of them, QUEST 2.0 and SUS survey, were also used as modified versions. IBM’s computer usability satisfaction questionnaires and USE questionnaires were only used as modified versions. Lastly, some studies proposed and conducted their own evaluation tools.

**Table 5:** Classification of articles by evaluation tool of subjective measures.

Evaluation tool	No. of articles	References
NASA-TLX	13	[52,55,58,76,143–145,148,152–154,157,163]
VAS	12	[52,58,76,143–145,148,150,152–154,157]
Proposed	8	[52,143,146,147,149,161,162,164]
Customized Questionnaire (Modified QUEST 2.0)	5	[52,143–145,148]
ATD PA Device Form	3	[52,143,144]
SUS survey	3	[55,154,163]
Customized Questionnaire (Modified IBM’s computer usability satisfaction questionnaires)	2	[158,159]
Customized Questionnaire (Modified SUS survey)	2	[149,160]
Customized Questionnaire (Modified USE Questionnaire)	2	[151,156]
QUEST 2.0	1	[153]

NASA-TLX is a popular mental workload assessment technique which relies on a multidimensional construct. It derives overall workload based on 6 subscales: mental demand, physical demand, temporal demand, performance, effort and frustration [166]. VAS is one of the methods of assessing a “feeling” [167]. It is usually conducted to assess the satisfaction of a system in BCI usability studies. ATD-PA device form and QUEST 2.0 are specialized subjective assessment tools used to evaluate the assistive devices. ATD-PA is a set of questionnaires used to assess the match quality experienced between the person and the assistive technology [168]. In BCI usability studies, only a set of 12 items, called ATD-PA device form, is usually utilized to ask users’ opinions of 12 aspects of using the proposed BCI system as an assistive device. QUEST 2.0 is an instrument used to evaluate users’ satisfaction with assistive technology. It contains 12 items rated on a 5-point satisfaction scale with regards to the device and services [169]. SUS survey and USE questionnaires are simple, yet effective tools used for assessing the usability of various products. SUS survey contains 10-item scale giving a global view of usability [170], and USE questionnaires contains 14-item scale consisting of four domains: satisfaction, ease of use, ease of learning, and usefulness [171]. IBM computer usability satisfaction questionnaires also measure user satisfaction with usability, but it is specialized on a computer system [172]. Questionnaire for current motivation (QCM) is the subjective assessment tool designed to measure users’ motivation with respect four motivational factors: mastery confidence, incompetence fear, challenge, and interest [173].

To find out the frequently used subjective measures in each type of task, we categorized studies by type of task and counted the number of studies separately. Among 19 spelling tasks, NASA-TLX and VAS were the most frequently used subjective evaluation tools in both closed copy tasks and self-managed tasks. In closed copy tasks, four studies proposed new subjective measures. Proposed measures varied from study to study. For example, Deravi et al. [149] evaluated aesthetic, attractiveness, cognitive workload, comfort, ease of use, effectiveness, functionality, helpfulness, operability, safety, and usefulness of system through their own developed set of questionnaires. Hohne & Tangermann [52] evaluated controllability, effectiveness, efficiency, and exhaustion; and Nam, Li, & Johnson [161] evaluated preference. Won et al. [164] evaluated comfort, and Nijboer et al. [162] did aesthetic, comfort, operability, and preference.

In movement control tasks, NASA-TLX and VAS were also most frequently used in all types of task. Among the seven studies of movement control tasks, only three studies did not use any subjective measures for usability evaluation of BCI [62,66,155]. In addition, Modified QUEST 2.0, SUS survey, and modified SUS surveys were used in the closed copying task and ATD PA device form was only used in closed self-managing tasks and QUEST 2.0 was only used in open tasks. Among nine studies using selection control tasks, seven studies used subjective measures, and one study proposed new measures in the closed copying task [149]. Only two studies did not use subjective measures in the selection control task experiments [64,155]. In the cognitive rehabilitation and brain painting tasks, all studies used subjective measures. One study using a brain painting task proposed a new measure in open task: exhaustion [52]. Among two studies using a mental task, only one study used subjective measures [147]. Weyand et al. [147] proposed the new measure “helpfulness of feedback”.

Overall, task accuracy and ITR were most frequently used for performance measures. The rest of the measures were related to brain activity (e.g., amplitude, latency), time dependent variables (e.g., task speed, task time, time for selection), and the difficulties of task completion (e.g., error rate, feasibility of finishing the task, task completion rate). Also, there were some studies that proposed new metrics for performance measures (e.g., effectiveness [152], efficiency [63,144], WS score [147]).

To find out the frequently used performance measure in each type of task, we categorized studies by type of task and counted the number of studies separately. Among 19 spelling task studies, only one study did not use performance measures to evaluate usability of BCI [58]. In both closed self-managed tasks and closed copy tasks, task accuracy and ITR were most frequently used as performance measures. Because there were more studies using a closed copy task than a closed self-managed task, performance measures, which were used in the copy task, except task accuracy and ITR, varied depending on study. In movement control tasks, all studies used performance measures. Task accuracy was most frequently used, whereas ITR was used in only one study [52]. One study of the brain painting task did not use performance measures [143].

Since the objective of this study was to establish the usability dimensions measured in BCI usability studies, we reorganized them in terms of usability dimensions. Table 6 presents a summary of 40 measured subjective usability dimensions. A preliminary inspection of Table 6 shows that the constructs of satisfaction, cognitive workload, and ease of use are most commonly measured in BCI usability studies. All of these measures were defined in the work of Han et al. [174] on the classification of performance and image/impression dimensions with slight variations.

**Table 6:** Frequency of subjective measures used in the reviewed articles.

Measures	References	Count	%
Satisfaction	[52,58,66,76,144–146,148,149,151–154,156–160]	18	58.06
Cognitive workload	[52,55,58,76,143–145,148,149,152–154,157,163]	14	45.16
Ease of use	[52,55,143–147,149–151,156,158,159,163]	14	45.16
Mental demand	[52,55,58,76,143–145,150,152,163]	10	32.26
Comfort	[52,143–145,149,158–160,162,164]	10	32.26
Effort	[52,55,58,76,143–145,152,163]	9	29.03
Frustration	[52,55,58,76,143–145,152,163]	9	29.03
Performance	[52,55,58,76,143–145,152,163]	9	29.03
Physical demand	[52,55,58,76,143–145,152,163]	9	29.03
Temporal demand	[52,55,58,76,143–145,152,163]	9	29.03
Efficiency	[52,58,76,143–145,154,163]	8	25.81
Learnability	[52,55,143–145,149,151,156,163]	9	29.03
Usefulness	[52,143,144,149,151,156,158,159]	8	25.81
Aesthetic	[52,143–145,149,162]	6	19.35
Helpfulness	[52,143–145,147,149]	6	19.35
Predictability	[52,55,143,144,149,163]	6	19.35
Effectiveness	[52,143–145,149]	5	16.13
Responsiveness	[52,143–145,160]	5	16.13
Safety	[52,143–145,149]	5	16.13
Adjustment	[52,143–145]	4	12.90
Enjoyment	[52,143,158,159]	4	12.90
Operability	[55,149,162,163]	4	12.90
Physical accommodation	[52,143–145]	4	12.90
Reliability	[52,143–145]	4	12.90
Adaptability	[52,143,144]	3	9.68
Complexity	[55,160,163]	3	9.68
Consistency	[55,149,163]	3	9.68
Exhaustion	[52,143,146]	3	9.68
Expected technology benefit	[52,143,144]	3	9.68
Familiarity	[52,143,144]	3	9.68
Preference	[58,161,162]	3	9.68
Privacy	[52,143,144]	3	9.68
Security	[52,143,144]	3	9.68
Willing to use	[55,62,149,163]	3	9.68
Functionality	[55,149,163]	2	6.45
Recommendability	[158,159]	2	6.45
Attractiveness	[149]	1	3.23
Clarity	[146]	1	3.23
Controllability	[143]	1	3.23
Mood	[153]	1	3.23

We reorganized performance measures (objective measures). Table 7 presents a summary of 21 performance measures. From Table 7, task accuracy and ITR are most commonly measured in BCI usability studies. The remaining performance measures varied depending on study.

**Table 7:** Frequency of performance measures used in the reviewed articles.

Measures	References	Count	%
Task accuracy	[52,62,63,65,66,76,144–146,148–150,152,154,156,157,160,161]	18	58.06
Information Transfer Rate	[52,55,76,144,145,150,152,157,161,163]	10	32.26
Classification accuracy	[147,151,155,162–164]	6	19.35
Amplitude	[150,157,161]	3	9.68
Task time	[64,156,160]	3	9.68
Error rate	[63,66]	2	6.45
Latency	[157,161]	2	6.45
Proposed metric of efficiency	[63,144]	2	6.45
Task speed	[62,65]	2	9.68
Throughput time	[65,76]	2	3.23
Abstentions	[63]	1	3.23
Errors	[64]	1	3.23
Hybrid system accuracy	[76]	1	3.23
Proposed metric of effectiveness	[58]	1	3.23
Real time to setup	[162]	1	3.23
System accuracy	[66]	1	3.23
Task completion rate	[65]	1	3.23
The feasibility of finishing the task	[160]	1	3.23
Time for correct selection	[154]	1	3.23
Time for selection	[76]	1	3.23
WS score	[147]	1	3.23

Upon review of the measures’ frequency in the collected articles the three core-constructs for the measurement of usability appear to be the following [175]:

- Efficiency: Degree to which the product is enabling the tasks to be performed in a quick, effective, and economical manner, or is hindering performance.
- Effectiveness: Accuracy and completeness with which specified users achieved specified goals in a particular environment.
- Satisfaction: The degree to which a product is giving contentment or making the user satisfied.

**Fig 5:**
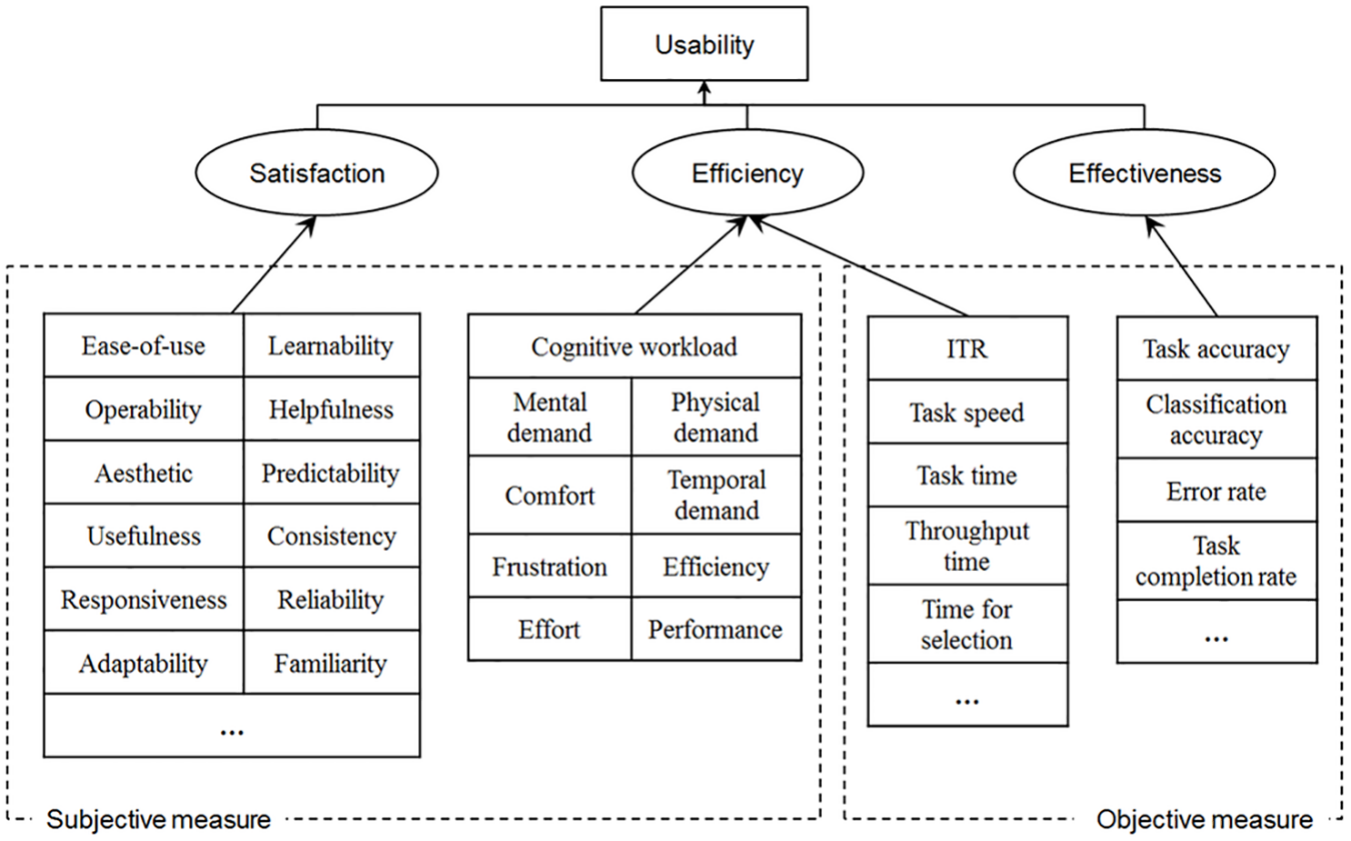
Usability dimensions for BCI systems.

Most subjective measures take into account Satisfaction and Efficiency. The metrics for evaluating cognitive workload by NASA-TLX were used for assessment of efficiency of BCI systems. Although various subjective measures were used and the definitions of each measure were different, the purpose of determining the usage of the measures except cognitive workload is to assess user’s satisfaction on BCI systems. Performance measures varied depending on study. An accuracy measure was typically used to assess the effectiveness of BCI systems. Among the collected articles, ITR was typically used to assess efficiency of BCI systems. Because the measures are related to how the user performs the task in a quick and effective manner, task speed time, throughput time, the feasibility of finishing the task, and time for selection can be involved in efficiency measures. A summary of usability dimensions is illustrated in Fig 5.

## Opportunities for further research

### Research question 1

After categorizing selected research studies in terms of the proposed taxonomy, three main issues were found from the current hybrid BCIs. First, few hybrid BCI studies were validated by actual target users such as disabled patients while others recruited healthy subjects to test the proposed system. To realize user-centered hybrid BCI systems, the actual target users should be involved in the evaluation of proposed systems, because the ultimate goal of the BCI system is to help severely disable people [34]. In addition to the recruitment issue, the sample size of current hybrid studies were less than 15 subjects in all but five studies [90,101,106,108,121], and these studies cannot guarantee duplicable results and they do not truly represent general target users. To address this issue, future research should consider having expected users involved from the system design stages to the experiment stages. The second issue is that the current hybrid BCI systems still rely on non-neurological signals, such as physiological or conventional external devices. Even though BCI systems can be applied to able-bodied persons, it cannot be denied that current BCI systems are mainly targeting severely disabled patients who cannot utilize traditional external devices such as eye trackers. Therefore, a further direction of hybrid BCI studies should incorporate a homogeneous hybrid BCI system. The last issue is that usability of the hybrid BCI was evaluated only in two research studies [76,136], and this issue was discussed in Study 2.

Despite aforementioned issues, there were some interesting findings uncovered after applying the proposed taxonomy to the selected studies. Firstly, there were a substantial amount of studies that used a single modality with multiple signatures in terms of brain signals, such as a combination of either visual-based SSEP and ERP or tactile-based SSEP and ERP. However, there was a lack of studies that utilized a single-signal signature with multi-modality such as a combination of either multi-modal SSEPs (e.g., tactile and visual-based SSEPs) or ERPs (tactile and auditory-based ERPs). Since both SSEP and ERP are the most reliable modalities should produce more optimal results.

### Research question 2

From the perspective of ergonomics design, BCI systems have several issues. First, standardization is difficult to apply in BCI systems. Since previous studies have focused on improvement in speed and accuracy of recognition methods (e.g., signature and classification algorithms), several basic principles used to increase performance of BCI systems in the confined environment over a certain level have been identified. For instance, support vector machines, dynamic classifiers, and combinations of classifiers are effective and powerful for synchronous BCI systems [19]. However, even though using the same mental task, classification algorithm, and measuring equipment, the performance and comfort of BCI systems can change according to the users’ characteristics (e.g., physical/cognitive disabilities, anthropometric traits) [176–179]. Also, due to users’ anthropometric traits being diverse (e.g., size and shape of users’ head), the fitness of BCI systems can change according to users [180,181]. The type and design of electrodes have significant influence on artifact signal [182]. In the case of ‘wet’ electrodes, the quality of signal can change according to participants’ head shape and size, hair type and length, and scalp properties [183]. Second, in BCI systems, it is needed to develop a new way of interaction for enhancing perspicuity and compatibility of BCI systems. UI responds to events triggered by users as they click the mouse and selecting the menu. Thus, it is an indispensable part of HCI for the advantage of perspicuity and compatibility, in terms of ease of operation. However, the way of interaction with BCI systems can be different from other conventional interfaces. Since detecting diverse mental tasks in BCI systems for intuitive interaction is difficult, it is limited to enhance ease-of-use in designing of BCI systems [177,184]. Especially, due to the purpose of BCI systems that aid disabled persons in controlling interfaces, the interaction between users and interfaces can be limited by users’ physical/cognitive disabilities. Thus, diverse functions are difficult to be applied in BCI systems. If control methods of diverse functions are designed by the limited way of interaction without considering perspicuity and compatibility, users will feel physical and mental fatigue quickly [176,177]. Finally, adaptability and scalability are scarce in BCI systems. BCI systems are difficult to be utilized in the users’ daily life. The set-up of BCI systems in daily life has limitations such as poor calibration due to environment, connection of sensors, and time consuming set-up of hardware and software [179,185]. Also, because BCI systems need space organization, the mobility of BCI systems is restricted [178,185]. Moreover, due to the lack of scalability of the BCI systems, it is difficult to interact with other existing software and devices [176,177,179].

Essentially, hybrid BCI systems have similar design issues as BCI systems. However, some issues can be more critical in hybrid BCI systems due to the complexity of hybrid BCI systems (e.g., more sensors of hybrid BCI systems, more sensory functions of users). First, physical or cognitive discomfort can be witnessed more than in BCI systems. Since hybrid BCI systems require more sensors to be attached, users can feel more physical discomfort. More gel for electrode caps and increased set-up time can cause more unpleasant feelings and annoy users [180,185]. Attaching more sensors can cause restricted users’ behavior and visual field. Attention allocation may be limited and managing distraction may increase [176]. Thus, users can feel fatigue more quickly. Second, technology acceptance or generalization of hybrid BCI systems can be less than BCI systems. Due to more cognitive actions involved, learnability and reliability of hybrid BCI systems can degenerate [186]. Also, due to hybrid BCI systems’ complexity, caregivers, friends or relatives should know how to control such complex systems without prior knowledge at the users’ home or bedside [177].

The solutions to the above issues can be related to usability evaluation for BCI and hybrid BCI systems. Previous studies of BCI and hybrid BCI usability have primarily focused on cognitive workload or performance (objective measures). Subjective measures can be helpful in identifying design problems from the perspective of HCI. Specifically, considering the usability measures in Fig 5, which are related to customization, compatibility, and scalability of BCI systems (e.g., predictability, adaptability, learnability, consistency, and familiarity), it is possible to define the problem and find the cause to develop or improve the BCI systems. Considering the proposed usability metrics in Fig 5, such as cognitive workload, learnability, adaptability, reliability, and ease-of-use, can be helpful for improving the complexity of hybrid BCI systems. Therefore, future research using those measures for usability evaluation is needed for solving ergonomics/HCI design issues of BCI and hybrid BCI systems.

The inspection method has not been conducted sufficiently for BCI and hBCI usability. Through utilizing the inspection method (e.g., heuristic evaluation, cognitive/pluralistic walkthrough, guideline checklist, etc.), it is possible to identify usability problems of the UI design in a detailed manner. The inspection method specifically involves evaluators (end-users or UI experts), and is conducted in context-of-use cases (typical user tasks), to provide feedback to the developers on the extent to which the interface is likely to be compatible with the intended users’ needs and preferences. Future research of usability evaluation for BCI and hybrid BCI systems is needed in order to identify and solve the aforementioned usability problems.

## Conclusions

We systematically reviewed and analyzed the current state-of-the-art hybrid BCI studies and proposed a clear and systematic taxonomy of hybrid BCIs with multiple taxonomic criteria. With this taxonomy, hybrid BCIs can be classified in terms of 1) the source of the signals, 2) the characteristics of the signal, and 3) the characteristics of operation in each system. Thus, BCI researchers, even those who new to the field, can easily understand the complex structure of the hybrid system at a glance. Furthermore, this review outlined the advantages and disadvantages of each hybrid BCI system in regards to what should be considered according to system environment, conditions, and target users.

The results in accordance with the proposed taxonomy show that many hybrid BCI studies (58%) utilized EEG signals with multi-signatures in combinations such as 1) SSEP and ERP, 2) SSEP and MI, and 3) ERP and MI. A quarter of the studies combined a brain signal with physiological signals such as EOG (8%), EMG (4%), and ECG signal (3%) to take the advantages of the higher signal-to-noise ratio of physiological signals in comparison to neurological signals, while comparable studies added an external device, such as an eye tracking system (15%), a joystick (3%), and a gyroscope (1%) to a BCI system for directional controls. In terms of the characteristics of the signal, most of the studies used modulated brain signals via operant conditioning with mental tasks, and stimulus evoked brain signals via selective attention were added to support or coincide with. For the stimulus modality, all of the reviewed studies applied visual and/or operant conditioning except three studies [42,89,119]. The most widely used brain signal signatures were visual-ERP, SSEVP, or MI. The proposed taxonomy also clarified the current research limitations for future research directions. Most of the previous studies did not evaluate the proposed hybrid BCI systems with real target users such as disable patients. Instead, the systems were tested with healthy participants in all but six research studies [76,82,88,91,116,125]. Of the experiments that were conducted with disabled patients in the hybrid BCI studies, all of those were case studies with small sample sizes.

The other issue found in this review was that current hybrid BCI research studies still highly rely on either visual stimulation or external devices that might not be possible to apply to some target user groups including severely disable patients. Also, we exhaustively reviewed recent literature on usability of BCIs. To identify the key evaluation dimensions of usability, we focused on task and measurement characteristics of BCI usability. We classified and summarized BCI usability studies according to task characteristics (type and description of task) and measurement characteristics (subjective and objective measures). Afterwards, we proposed usability dimensions for BCI and hybrid BCI systems with recommendations for further research.

We found that previous studies of BCI and hybrid BCI usability have primarily focused on evaluating performance and cognitive workload of systems. From the results of classifications, the three core-constructs for the measurement of usability appear to be: Satisfaction, effectiveness, and efficiency. In satisfaction, all involved metrics are subjective measures. Those measures (e.g., ease-of-use, learnability, operability, helpfulness, etc.) are usually rated on a 5 or 7 point likert scale. All metrics in effectiveness are objective measures. The measures of effectiveness are related to accuracy and completeness with which specified users achieved specified goals in a particular environment. In efficiency, there are both of subjective and objective measures. Subjective measures in efficiency are related to users’ cognitive workload. NASA-TLX has been widely used to evaluate users’ cognitive workload. Objective measures in efficiency are related to time and speed. Those measures aim to evaluate the degree to which the system is enabling the task to be performed in a quick, effective, and economical manner, or if it is hindering performance. Utilizing those usability dimensions can help researchers and practitioners understand BCI studies related to usability evaluation, and choose proper metrics for usability evaluation of BCI and hybrid BCI systems.

Opportunities for further research were discussed in this study. Most of the previous studies have focused on cognitive workload and performance of systems. Thus, studies focused on subjective measures, especially with regards to the inspection method, could be conducted in the future. Considering the proposed usability measures, it is possible to identify and solve design issues of BCI and hybrid BCI systems. Specifically, to enhance customization, compatibility, and scalability of BCI systems, predictability, adaptability, learnability, consistency, and familiarity should be selected for usability evaluation. Also, considering proposed usability metrics such as cognitive workload, learnability, adaptability, reliability, and ease-of-use can be helpful for improving the complexity of hybrid BCI systems. Therefore, suggestions for future research directions in this study can be helpful in establishing research directions and gaining insight in how to solve ergonomics and HCI design issues surrounding BCI and hybrid BCI systems.

## Supporting information

S1 File PRISMA checklist. (PDF)
